# Impediments to countering racist pseudoscience

**DOI:** 10.1017/ehs.2025.10012

**Published:** 2025-07-22

**Authors:** Kevin N. Lala, Gillian Brown, Kalyani Twyman, Marcus W. Feldman

**Affiliations:** 1 School of Biology, University of St Andrews, St Andrews, UK; 2 School of Psychology & Neuroscience, University of St Andrews, St Andrews, UK; 3 Department of Biology, Stanford University, Stanford, CA, USA

**Keywords:** race, racism, genetic determinism, inheritance, teaching

## Abstract

Although virtually all academics who study human ‘race’ agree that it is a social construct, members of the general public still commonly regard ‘race’ as a biological property (i.e. they think that ‘races’ are genetically distinct). Even though empirical data from genetics and other fields do not support biological conceptions of race, this erroneous viewpoint is widely held, suggesting that there are impediments to effective communication of the relevant science. Here, we suggest five such impediments: (1) belief in genetic determinism, together with an over-reliance on an essentialist view of human groups, (2) overly simplistic interpretation of biological inheritance, (3) belief in the naturalistic fallacy and the associated naturalization of non-biological variation among racialized groups, (4) failure of the academic and educational communities to take responsibility for teaching the science of ‘race’ and racism, and (5) apologism towards racist founders of academic fields, including the evolutionary sciences. We address how and why each of these factors supports the spread of racism and suggest strategies for containing this spread.

## Graphical abstract

**Figure:**
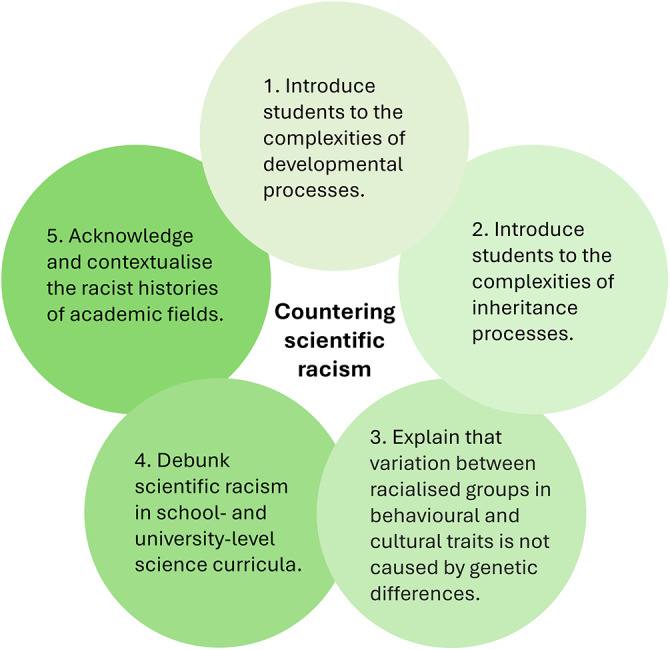

## Social media summary

We discuss impediments to countering racist pseudoscience and present five potential solutions for scientists and teachers

## 1. Introduction

The relationship between science and racism is complex. On the one hand, for at least three centuries, eminent Western scientists have devised racist classification schemes or have actively promoted and reinforced racist views (Farber, 2011; Painter, 2010; Sussman, 2014); these scientists have included important contributors to the biological sciences such as Carl Linnaeus, Georges Cuvier, Francis Galton, and Ronald Fisher. On the other hand, contemporary science provides the tools to debunk racist ideas, with incontrovertible empirical evidence that ‘racialized groups’ (i.e. people who are designated by society as members of a ‘race’) cannot be distinguished on the basis of shared sets of genes, simple biological markers or isolated genetic lineages (e.g. Hunley et al., 2016; Rosenberg et al., 2002, 2005; reviewed by Templeton, 2013; Graves & Goodman, 2022). Because the evolutionary human sciences focus on both human universals and human diversity (Brown et al., 2011; Brown & Lala, 2024), they are uniquely placed to play a role in repudiating inaccurate portrayals of humanity. However, combatting scientific racism also entails reflection on how the concept of human ‘races’ influenced the foundation of the evolutionary human sciences and how it continues to infiltrate and shape this field. The aim of this article is to identify some impediments to countering scientific racism operating within academia, along with some practical solutions for overcoming them.

Among academics who study ‘race’ and racism, there is a consensus that ‘race’ is a social construct (Wagner et al., 2017) rather than a reflection of physical reality. These authorities, as well as many people outside of academia, would accept a definition of racism along the lines of ‘systematic discrimination by a powerful individual or institution against individuals based on their perceived membership in a socially defined racial group’ (Graves & Goodman, 2022, p. 18). This definition has the advantage that it captures how racism can become embedded within a range of societal structures, including academia itself. Yet, these definitions of race and racism are not universally accepted. Despite decades of teaching about the meaning of ‘race’, a disturbingly large fraction of people continues to view it as a biological concept (Condit, 2007; Donovan, 2015; Royal, 2023). Individuals with stronger racial prejudice have been found to be more likely to believe that biological ‘races’ exist and to attribute behavioural traits to genes (e.g. Jayaratne et al., 2006; Singer et al., 2010). In addition, some subsections of the public embrace a narrower definition of racism that equates it with racial prejudice on the part of ‘evil’ people with extreme views, while eschewing or actively denying the existence of institutional or structural racism (Graves & Goodman, 2022). This position has been adopted by many right-wing and white supremacist elements.

Historically, there have been scientists who contributed to the racism in society by lending it credence and authority (reviewed by Bird et al., 2024; Kevles, 1985; Sussman, 2014). Biological science reified folk ideas of ‘race’ that stemmed from ethnocentrism and xenophobia by giving them a ‘scientific’ underpinning (Fredrickson, 2002/2015; Painter, 2010). *Scientific racism* is the (false) belief that the human species is divided into biologically distinct taxa or ‘races’, and/or that empirical evidence exists to support or justify racial discrimination, racial inferiority, or racial superiority. There is increasing awareness that legitimate mainstream science, particularly in the field of human genetics, is regularly being coopted and distorted by the far right to promote extremist views (Carlson et al., 2022; Panofsky et al., 2024). As described below, scientific racism is not just a relic from the distant past but continues to exist in contemporary science (Lala & Feldman, 2024; Sear, 2021).

## 2. Scientific racism in the evolutionary human sciences

The evolutionary human sciences emerged as a field of study in the 1970s in the aftermath of the ‘race and IQ’ and ‘human sociobiology’ debates (Brown et al., 2011). In 1969, Berkeley psychologist Arthur Jensen published his now infamous article ‘How much can we boost IQ and scholastic achievement?’ in which he claimed, erroneously, that high heritability would make it impossible for societal interventions to reduce the reported IQ difference between White and Black Americans. This publication, and the emergence a few years later of human sociobiology (Wilson, 1975, 1978), reinvigorated the ‘race’ debate, contributing to the combative intellectual environment in which the evolutionary human sciences emerged (Segerstråle, 2000). The controversy has persisted for decades, further fuelled by the publication of Herrnstein and Murray’s (1996) ‘The Bell Curve’, which sanitized but still promoted Jensen’s claims. Indeed, similar claims about ‘race’ and cognitive ability continue to be made (e.g. Ashraf & Galor, 2013; Galor, 2022), with the academic literature tarnished by poor-quality research on IQ and educational attainment (reviewed by Sear, 2022).

Faced with these controversies, the field of *evolutionary psychology* took steps to separate itself from human sociobiology (Brown & Lala, 2024). Whether or not it was a deliberate strategy, evolutionary psychologists emphasized *universal* evolved psychological mechanisms that humans are assumed to possess, which allowed them to evade the issue of racial differences in intelligence, cognition, and educational success. The evolutionary psychology approach does acknowledge variation in behaviour in response to specific environmental or internal inputs (e.g. through condition-dependent strategies), but assumes that this variation is generated by universally shared structure in the human brain (except in the case of differences between the sexes; see below). A second subfield of the evolutionary human sciences, *human behavioural ecology*, side-stepped the controversy in a different way, by adopting a functional perspective focused on whether human behavioural strategies are adaptive across a range of ecological and social conditions. This tactic allowed the causes of differences between groups to remain unspecified, although the focus on behavioural diversity across small-scale societies, which often live in close proximity to each other, suggests that human behavioural ecology does not view genetic variation between populations to be the main factor that might account for this diversity.

One subfield of the evolutionary human sciences that did address the issue of ‘race’ directly is *cultural evolution*, for which the original impetus was Jensen’s (1969) article and the contemporaneous racist advocacy of eugenicist William Shockley (Brinitzer, 2024). The founders of cultural evolution theory, Luca Cavalli-Sforza and Marcus Feldman, were disturbed by the publicity garnered by the writings and speeches of Jensen and Shockley and set out to demonstrate why these claims were false by developing new statistical measures of the inheritance of traits that took the effects of cultural transmission into account (e.g. Cavalli-Sforza & Feldman, 1973; Feldman & Cavalli-Sforza, 1976). In recent times, however, the subfield of cultural evolution has largely focused on other questions, such as the evolution of cooperation, language, and learning strategies (Brown et al., 2011). The related subfield of *gene–culture coevolution* examines the evolutionary interactions between cultural activities and genetic variation, providing cultural explanations for the distribution of variation in some genes (e.g. alleles that allow lactose absorption in adulthood in pastoralist societies). The evidence emerging for gene–culture coevolution *counters* the idea that humans can be assigned to ‘races’ (Lala & Feldman, 2024). Thus, although the evolutionary human sciences began in the midst of controversies over ‘race’, the topic is not currently a major focus within the field.

Racist claims are nearly always ‘evolutionary’ in character and clearly concern the ‘human sciences’. As a consequence, racist pseudoscientific claims tend to appear in scientifically questionable venues on the fringes of the evolutionary human sciences and are occasionally reported at scientific meetings and sometimes in academic journals (reviewed by Sear, 2021, 2022). Since the advent of the evolutionary human sciences, there has been a constant stream of racist articles that are supposedly built on empirical research, but do not follow standard scientific methods or are based on flawed data, biased assumptions, or spurious arguments. Although many researchers have sought to counter these developments (e.g. Feldman, 2014; Feldman & Ramachandran, 2018; Henrich, 2016; Lala & Feldman, 2024; Sear, 2021, 2022), the persistence of scientific racism remains a serious concern. There would appear to be something alluring about the existence of meaningful genetic differences between human ‘races’, an observation that Lala and Feldman (2024) label ‘the fallacious intuitive argument’. However, researchers across many academic disciplines have not always acknowledged, nor challenged, scientific racism, even within their own field. Here, we discuss possible hurdles to the effective deterrence of scientific racism.

## 3. Five impediments to countering scientific racism

**Figure 1.**
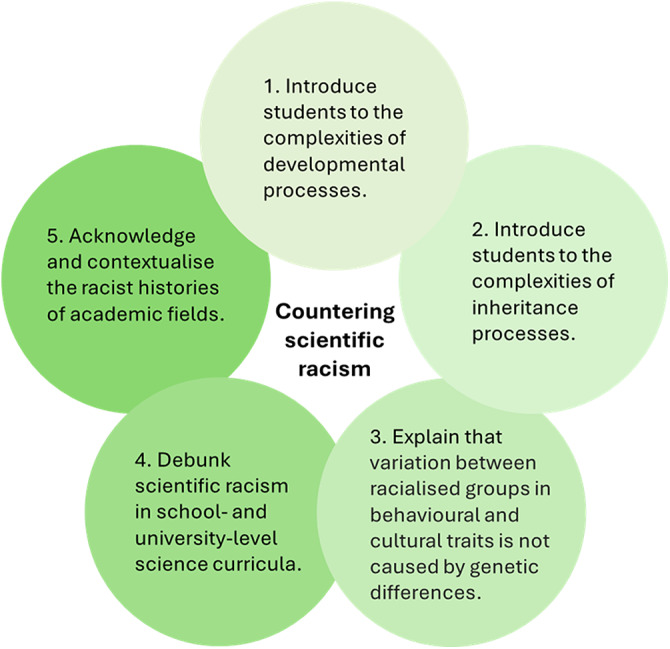
Five approaches to countering racist pseudoscience.

There may be substantial and persistent phenotypic differences between racialized groups, for instance, in disease incidence, sporting performance, or IQ test measures (Graves & Goodman, 2022). Unfortunately, people often jump to the conclusion that such disparities reflect genetic differences between racialized groups. Although this inference is incorrect, such misunderstandings are difficult to counter, particularly as controversial or negatively valanced information appears to spread faster than uncontroversial or positively valanced information (e.g. Jasser et al., 2022; Youngblood et al., 2023). Balanced arguments and cautious explanations of scientific findings are often less likely to receive attention from journalists or be shared on social media platforms than sensational but erroneous claims (Panofsky et al., 2024). In addition, some people adopt and promote racist views for political, religious, and economic reasons (Fredrickson, 2002/2015; Saini, 2019), or because of structural features of society, such as the racial classifications used in official censuses, or by police forces and judiciary (Guevara et al., 2023). Although such elements are likely important, here we focus on impediments to countering scientific racism that arise in academic settings, or that involve the dissemination of scientific findings. Below, we discuss five factors that may counter, undermine or block the effective communication of accurate science related to ‘race’ and racism, and thereby inadvertently promote racist pseudoscience. These factors are *genetic determinism, simplistic interpretations of inheritance, the naturalistic fallacy, a failure to teach the relevant science*, and *apologism towards racist founders and leaders*. For each impediment, we suggest possible solutions (summarized in Table 1 and Figure 1).

**Table 1.** Impediments to racist pseudoscience and potential practical solutions

Impediment to racist pseudoscience	Potential practical solution
Belief in genetic determinism	At high school and university levels, students should be introduced to the complexities of human development. Explanations for the development of human traits should emphasize gene–environment interactions and the complexity of underlying causation.
Overly simplistic conceptions of biological inheritance	At high school and university levels, students would benefit from being introduced to the complexities of biological and other types of inheritance and to the difficulties involved in inference of causation of human traits.
Belief in the naturalistic fallacy and associated naturalization of non-biological variation between groups	Evolutionary researchers should be more explicit in eschewing the naturalistic fallacy and stressing that variation between racialized groups in behavioural and cultural traits is not caused by genetic differences.
A failure of relevant scientific disciplines to take responsibility for teaching the science of ‘race’ and racism	The relevant knowledge that exposes racist claims to be false needs to be incorporated into science curricula, and interdisciplinary approaches to teaching about ‘race’ and racism encouraged.
The self-promotion of academic fields, and apologism towards racist founders and leaders	The historical background to academic fields, including racist histories, should be openly acknowledged and appropriately contextualized.

### 3.1. Impediment 1: genetic determinism

Attribution of group differences to genetics provides simple, easy-to-understand explanations that sound credible to a general public that is regularly fed a diet of ‘gene-for-X’ explanations in the media (Condit, 2007; Moore et al., 2025). This ‘gene for’ language is commonly misunderstood to imply *genetic determinism* (e.g. Bates et al., 2003; Condit & Parrott, 2004; Nelkin & Lindee, 2004), namely the belief that phenotypic traits are exclusively, or predominantly, caused by genotypes. Although many scientists and journalists may be prone to overhyping scientific findings, this tendency can be particularly problematic in the field of genetics, where a genetic association is rarely qualified as just one of many possible causal factors and is often framed as a ‘breakthrough’ that will lead to a new cure or treatment (Condit, 2007). Where scientists discover significant gene–environment interactions, it is the genes that are usually highlighted, with less emphasis placed on the role of the environment (Dar-Nimrod & Heine, 2011). Similarly, when genome-wide association studies (GWAS) are published, the headline commonly states that ‘genes affecting trait Y have been discovered’, with little prominence given to the often-small amount of trait variance explained by DNA variants. News articles frequently misrepresent heritability estimates in humans as a measure of the relative importance of genes in causing a trait, as opposed to referring to *variation in* a trait, and in this way the public is given the impression that genes are the sole or major cause of many human conditions (Moore et al., 2025). Belief in genetic determinism, and essentialist reasoning, has been reported to be common in many countries and cultures (Dar-Nimrod & Heine, 2011; Gericke et al., 2022; Keller, 2005) and recognized as ‘a societal problem because it has the potential to foster intolerant attitudes’ (Gericke et al., 2017, p. 1223; 2022).

Unfortunately, a basic education in genetics may not always help students to understand the complex interplay between genes and environments that produce human traits, nor does it encourage them to regard populations as aggregates of genetically varying individuals who differ in a continuous manner in a variety of quantitative characters (Donovan, 2022; Jamieson & Radick, 2017; Schmid et al., 2022; Stern & Kampourakis, 2017; Stern et al., 2023). On the contrary, studies have found that the teaching of simple model systems widely used to introduce students to Mendelian genetics (e.g. ‘blue’ versus ‘brown’ eyes), which are often portrayed in terms of direct genotype-to-phenotype mappings, can inadvertently encourage both genetic determinism and *genetic essentialism* (defined as thinking in terms of genetic ‘types’) (Donovan, 2017; Donovan et al., 2024a; Jamieson & Radick, 2017; Morin-Chassé, 2014; Schmid et al., 2022). This finding has led to the concern that undergraduate biology curricula are not aligned with current genomic knowledge, because the tests and assessments used by genetics instructors rarely assess concepts integral to multifactorial inheritance (Schmid et al., 2022). Encouragingly, where genes are no longer presented to students as ‘what inheritance is all about’ with everything else relegated to negligible supporting roles, and when students are instead taught the importance of multifactorial causation and environmental contingency, evidence suggests that a genetics education can ‘inoculate’ students against genetic determinism (Donovan et al., 2019a, 2021; Dougherty, 2009; Jamieson & Radick, 2017; Radick, 2016). Unfortunately, resistance to introducing these progressive teaching methods remains.

The wider issue here is that, for effective ‘inoculation’, students need, at some level, to understand the complexities of human development (Dougherty, 2009; Govindaraju & Goldstein, 2025; Moore et al., 2025; Schmid et al., 2022). Few human phenotypes are explained by single genes, and most are not only polygenic but shaped by a wide variety of other environmental and developmental factors (Barresi & Gilbert, 2023; Gilbert & Epel, 2015). The convenient shorthand of thinking of genes as having dedicated functions gives a false impression, as virtually all human genes have multiple functions and are expressed in multiple tissues. Although the simple ‘genotype-to-phenotype mapping’ may be an appealing metaphor, in reality there are many intervening steps in the path from DNA sequences to phenotypic characters, and any such ‘mapping’ is highly nonlinear and contingent on the individual’s internal and external environment (Bateson & Martin, 1999; Lala et al., 2024; Moore et al., 2025). A challenge to presenting a complex, but more accurate, description of the development of traits is that simplified accounts are often easier to understand. Simple heuristics allow complex scenarios to be interpreted and decisions to be made based on limited information about how the world seems to work (Gigerenzer & Brighton, 2011). As a result, this apparent simplicity can lead to stereotyping and an over-reliance on essentialist thinking, which can inadvertently promote racism and other forms of discrimination, including sexism (see Box 1).

Nonetheless, compelling recommendations, which we endorse, have been made for modernizing, complexifying, or ‘inverting’ (to focus on more representative complex cases first) biology curricula to enhance scientific understanding and reduce inappropriate essentialist and deterministic inference (e.g. Donovan et al., 2019a, 2021; Dougherty, 2009; Jamieson & Radick, 2017; Radick, 2016; Schmid et al., 2022).

**Solution:** *At high school and university levels, students should be introduced to the complexities of human development. Scientists should add nuance to explanations for the development of human traits by explaining gene–environment interactions and the complexity underlying causation.*

Box 1: Some parallels between racism and sexism

Just as an over-emphasis on genetic determinism is an impediment to anti-racism, it is also a barrier to dispelling sexism. In considering phenotypic differences between men and women, genes are often assumed to play a dominant causal role, including via downstream processes such as exposure to gonadal hormones during early life (Baron-Cohen, 2003; Herbert, 2015). Whereas genes contribute significantly to human sexual differentiation, any suggestion of strictly dichotomous sexes has been dispelled by researchers who have documented variability in these developmental processes (DuBois & Shattuck-Heidorn, 2021; McLaughlin et al., 2023). Gender differences, and similarities, in human behaviour and cognition, involve a complex interplay between both physiological and socialisation processes (Wood & Eagly, 2012). As manifest in some instances of racism, the over-attribution of these characteristics to genes can become associated with sexism and prejudice. For instance, individuals who attribute gender differences in behavioural and cognitive traits to genes are more likely to endorse sexist attitudes (e.g. Lee et al., 2020). Within the evolutionary human sciences, evolutionary psychologists have devoted considerable attention to men’s and women’s behaviours, frequently assuming that selection has favoured different psychological mechanisms. However, simplistic assumptions or conclusions along these lines risk justifying male superiority and acceptance of inequality.

Social and cultural processes dictate the roles that men and women are expected to adopt in a society, and socialization teaches children what is expected from them in terms of gender-specific behaviour, attitudes, skills, choices, and responsibilities (Wood & Eagly, 2012). Hence, the suggestion that this division of labour between the sexes is ‘natural’ or ‘inevitable’ is highly contentious. Even in Western societies, where levels of gender equality have generally increased over time, gender-based stereotyping has remained strong, although the content of these stereotypes has changed to some extent (e.g. Eagly et al., 2020). People also commonly assume, as they do for racial groups, that the categories of ‘man’ and ‘woman’ possess underlying ‘essences’, namely fixed, inborn, biologically defined attributes that cause the psychological traits of individuals in those groups (Dar-Nimrod & Heine, 2011; Donovan, 2015). Essentialist thinking provides a short cut for making predictions about the world, but with the drawback that it can promote simplistic stereotypes (Bastian & Haslam, 2006; Dar-Nimrod & Heine, 2011). Science textbooks often reinforce gender-based stereotypes (Donovan et al., 2024b), and experimental exposure to fictional material that endorses gender essentialism is reported to decrease support for gender equality (e.g. Donovan et al., 2019b; Wilton et al., 2019). Therefore, as with racism, education that challenges genetic determinism and gender essentialism could potentially reduce gender inequality and discrimination.

### 3.2. Impediment 2: overly simplistic conceptions of biological inheritance

A related concern is an overly simplistic conception of heredity that attributes the inheritance of traits, and differences in traits, to genes and genetic variation. In stark contrast to this simplistic understanding, recent years have witnessed an explosion of scientific interest in, and experimental evidence for, diverse forms of extra-genetic inheritance that contribute to parent–offspring similarity (Bonduriansky & Day, 2018; Cavalli-Sforza & Feldman, 1981; Lala et al., 2024). A wide array of resources is now known to be passed across the generations and to influence the phenotype, including epigenetic marks, symbionts, hormones, nutrients, antibodies, prions, cultural knowledge, ecological legacies, and more. There is no question that this multifaceted inheritance applies to humans. Human biological inheritance has epigenetic, somatic, cultural, and ecological components, which can cause variation between groups and perpetuate them across generations (Ivey Henry et al., 2023; Lala & Feldman, 2024; Lala et al., 2024). Yet for human traits such as disease risk, which may concentrate in families, there is a persistent tendency to assume that genetic inheritance is the main causal factor. Genes certainly contribute to explaining some of the visible phenotypes that are frequently referred to in racial classifications (e.g. skin pigmentation) (Jablonski, 2021; Quillen et al., 2019). However, most traits that are assumed to differ between racialized groups have a much more complicated aetiology.

Simplistic conceptions of inheritance fail to consider a broad range of inheritance processes (Lala & Feldman, 2024). ‘Epigenetic inheritance’ includes the transmission across generations of molecular markers that affect whether specific genes are expressed (e.g. via the attachment of histone proteins to cellular DNA; Anastasiadi et al., 2021). Although the conclusive controlled experiments that confirm epigenetic inheritance in other species are unethical, our own substantial circumstantial evidence suggests that epigenetic inheritance contributes to the inheritance of disease, stress, body weight and other traits in humans (Heard & Martienssen, 2014; Horsthemke, 2018; Jablonka & Lamb, 2014). Human babies also inherit some of their mother’s symbiotic bacteria during childbirth and in breast milk, as well as many other nutrients and resources (‘somatic inheritance’). Children inherit prejudices, beliefs, attitudes, and aspirations from parents and relatives (‘cultural inheritance’). They are also born into the environments of their parents, inheriting birth locations that vary in their quality of housing, childcare, schooling, pollutants, and other factors (‘ecological inheritance’). Epigenetic, somatic, cultural, and ecological inheritances affect a wide variety of human phenotypes, and all of these factors contribute to (often reliably inherited) phenotypic variation between racialized groups (Lala & Feldman, 2024).

An example of how simplified accounts of inheritance can lead to support for racism is provided by the debates around ‘race’ and scholastic achievement. Although numerous data sets have documented differences in the average levels of scholastic achievement between racialized groups in countries such as the USA (e.g. Kao & Thompson, 2003; Silverman et al., 2023), the persistence of these differences over time is not due to genetic transmission, as no such genetic differences have been found (Guevara et al., 2023). In this case, the racial variation arises because humans have constructed ‘inequitable niches’ that persist over time through the legacies of inherited wealth and power, inherited norms and institutions, and inherited environments that vary in their amenities and opportunities (Ivey Henry et al., 2023; Lala & Feldman, 2024). Variation in scholastic performance is primarily the product of different life experiences, including racism and discrimination within schools and other social settings (Turetsky et al., 2021; Warikoo & Carter, 2009). Similar critiques apply to simplistic accounts of variation in disease incidence or sporting achievements among racialized groups (Graves & Goodman, 2022; Ivey Henry et al., 2023). Detailed studies of extragenetic inheritance are necessary to understand how variation between racialized groups can emerge and persist over generations.

Some studies have attempted to downplay the importance of environmental influences on social status by suggesting that cultural variables are themselves genetically determined (e.g. Clark, 2023; for a rebuttal of this study, see Benning et al., 2024). This work claims that cultural variation is driven by genetic differences in parental ‘quality’, with genetically superior parents producing higher-quality offspring both through the genetic inheritance of intelligence and through their superior genes creating better nurturing environments. However, that social status persists across generations and has not decreased over time, despite efforts to improve social mobility, does not mean that genetic inheritance explains persistence of social status. The ‘genetification’ of culture has a sinister side. If all facets of an individual’s life were determined by genetics, rather than social experience and other extra-genetically transmitted factors, then inequities in power and wealth could be regarded as natural and inevitable (Saini, 2019). However, this interpretation entails a model of causation that the evolutionary human sciences have long rejected. For instance, a major conclusion of contemporary gene–culture coevolution research is that the interactions between genes and culture are bidirectional, and that the causes of cultural variation cannot be reduced to genetics (Brown et al., 2011; Henrich, 2016).

**Solution:** *At high school and university levels, students would benefit from being introduced to the complexities of biological and other types of inheritance and to the difficulties involved in inference of causation of human traits.*

### 3.3. Impediment 3: belief in the naturalistic fallacy and associated naturalization of non-biological variation among racialized groups

The *naturalistic fallacy* is the assumption that what is ‘natural’ is ‘good’. In the context of the evolutionary human sciences, the fallacy usually pertains to the claim that features of human behaviour or cognition that are thought to have evolved (e.g. male aggression) are desirable or inevitable. Although this assumption has been widely criticized (e.g. Curry, 2006; Wilson et al., 2003), it remains prevalent among the general public (Nelkin & Lindee, 2004) and in some academic literature, including the human sciences (Curry, 2006; Wilson et al., 2003), where evolution is perceived to naturalize contemporary political and economic relations between racialized groups, thus reinforcing genetic determinism.

In the late nineteenth and early twentieth centuries, the popularity of eugenics and social Darwinism led to the belief that the superior power and wealth of Western nations compared to the rest of the world reflected in-built differences in the psychology and abilities of different ‘races’ (Brown et al., 2011; Kevles, 1985; Levine, 2010). Likewise, the growth of Western nations as industrialized global powers has often been attributed to the intrinsic (biological and cultural) merits of these nations (Ashraf & Galor, 2013; MacMaster, 2001), rather than to colonialism and the transatlantic slave trade (Inikori, 2020). Even today, differences in wealth, power and success among racialized groups are frequently linked to the possession of more-or-less ‘effective’ cultural values and institutions, rather than to historical causes and their resultant contemporary structures. For instance, differences between African and European Americans are misattributed to ‘shortcomings’ of the former, deemed to reflect inborn or natural inferiorities, rather than to the legacies of slavery, racism, and discrimination (Kendi, 2019). The converse stereotype that Asian Americans prosper due to cultural values of hard work and education is also problematic, as it also encompasses essentialist assumptions about intrinsic difference.

Scholars of the evolutionary human sciences have a particular responsibility when reporting their research to avoid reinforcing hierarchical views of behaviour or culture. Evolutionary research often makes inferences from measured, or assumed, variation in the estimated ‘fitness’ of individual- or group-level traits. Although this approach should not entail attaching intrinsic value judgements to more-or-less ‘fit’ characteristics, where such traits concern human behaviour, cognition, or culture, there is a particular danger that ‘fit’ traits (i.e. those that are readily propagating) will be assumed to be ‘good’ (i.e. intrinsically meritorious). Unhealthy eating or drug use are traits that exemplify this important distinction. In the cultural evolution literature, it is easy to slip into regarding increased technological complexity as ‘inherently superior’ or ‘more evolved’, feeding a dangerous progressive narrative of human evolution. Similarly, care should be taken when interpreting data from contemporary small-scale societies to avoid inappropriately equating these populations with ‘ancestral’ states (Paige & French, 2020). Palaeoanthropology has employed many progress narratives to reinforce racist and colonialist hierarchies (Athreya & Ackermann, 2019). Such narratives might lend credence to simplistic accounts based on genetic determinism. For these reasons, it is imperative that evolutionary explanations for human behaviour, even those emphasizing cultural evolution, stress that evolution does not attribute value judgements to evolved characters, and that cultural variation between groups is not caused by underlying genetic differences.

**Solution**: *Evolutionary researchers should be more explicit in eschewing the naturalistic fallacy and stressing that variation among racialized groups in behavioural and cultural traits is not caused by genetic differences.*

### 3.4. Impediment 4: a failure of relevant scientific disciplines to take responsibility for teaching the science of race and racism

According to the UNESCO statement on ‘race’ (1952, p. 5), ‘Race hatred and conflict thrive on scientifically false ideas and are nourished by ignorance. In order to show up these errors of fact and reasoning… we must turn to the means and methods of education, science and culture.’ Higher levels of education are associated with lower levels of racial prejudice (Jayaratne et al., 2006), which implies that education, particularly biological education, may be a key ‘weapon’ against racism. As mentioned above, an education in genetics does not preclude emergence of belief in genetic determinism; however, there is evidence that a suitable (i.e. ‘holistic genetics’) education has a high probability of reducing belief in genetic essentialism and thus reducing racial prejudice (Donovan, 2022; Stern & Kampourakis, 2017).

The forging of the modern evolutionary synthesis in the twentieth century established a Darwinian perspective on evolution grounded in theoretical population genetics, and many of its architects were not just scientists but public intellectuals who were conscious of the social implications of the synthesis (Farber, 2011). The Holocaust triggered a rejection of eugenics within the sciences, and, by the 1960s, textbooks taught that racial categories held little to no biological meaning (Farber, 2011). Some leading evolutionary biologists, including Theodosius Dobzhansky and Ernst Mayr, were overtly critical of aspects of scientific racism, such as that race mixing would inevitably have detrimental effects (although that did not prevent Dobzhansky from believing that races are biologically real). At the same time, anthropologists exposed the myth of racial categories and the relativity of cultural values (Farber, 2011). Likewise, the rise of behaviourism in psychology moved the field away from explaining differences between people by recourse to human ‘instincts’ to studying how learning and the environment affect individuals’ behaviour and cognition (Brown et al., 2011). However, the last 50 years has witnessed changes in the teaching of this topic.

Today, although the issues surrounding ‘race’ and racism continue to be taught by anthropologists, psychologists, and social scientists (e.g. Bird et al., 2024; Goodman, 2017; Zuberi et al., 2015), biologists appear to have largely eschewed this topic. Several researchers have suggested that contemporary undergraduate biology courses do not adequately equip students to understand the complexities of human biology or guard against essentialist thinking (Donovan et al., 2019a, 2021; Dougherty, 2009; Hennessey and Freeman, 2024; Moore et al., 2025; Schmid et al., 2022), although Donovan (2015, p. 1092) detects ‘little evidence that contemporary biology textbooks challenge stereotypical racial beliefs that are based in biological thinking’. Indeed, our strong impression is that, in both North America and Europe, ‘race’ and racism rarely even feature in biology curricula (see also Guevara et al., 2023). This deficiency has created a vacuum wherein well-funded white supremacist groups are propagating pseudoscientific arguments, necessitating the intervention of qualified scientists to expose their dangerous fallacies (Panofsky et al., 2021).

As racism is a major societal issue, there are strong grounds for advocating that ‘race’ and racism should be part of science curricula at both school and university level. A robust education, and particularly a more holistic view of genetics, could ‘arm’ students against developing prejudicial views, give students the ability to see through racist pseudoscience, and teach them how to react when encountering it (Donovan et al., 2019a; Jamieson & Radick, 2017). Currently, students are rarely taught that genetic essentialism has been widely discredited (Dar-Nimrod & Heine, 2011; Donovan, 2015; Moore et al., 2025). They may only encounter the intersection of ‘race’ and genetics within science curricula in the context of racial variation in the prevalence of some genetic diseases (Donovan, 2015, 2022; Morning, 2011), which, unfortunately, may serve to increase belief in genetic essentialism (Donovan, 2017). Indeed, there have been calls to eradicate the misleading reference to ‘race’ from medical school curricula (e.g. Degife et al., 2021), as well as calls to incorporate the topic of racism and to integrate anti-racist principles into psychology, biology and anthropology curricula (e.g. Cronin et al., 2021; Gupta & Stoolman, 2022; O’Connor & Robbins, 2024). In addition to teaching how human genetic data are inconsistent with the existence of racial divisions, this education could acknowledge the role of scientists themselves in the development of racist thought and the limitations of the historically Eurocentric viewpoint.

**Solution:** *The relevant knowledge that exposes racist claims to be false needs to be incorporated into science curricula, and interdisciplinary approaches to teaching about ‘race’ and racism encouraged.*

### 3.5. Impediment 5: the self-promotion of academic fields, and apologism towards racist founders and leaders

Reticence to teach science that exposes biological ‘race’ as a myth may arise from scientists’ embarrassment about the histories of their fields. For instance, Steven Jay Gould (2000, p. 513) described evolutionary biology’s suppression of the history of its association with racism and eugenics as ‘a conspiracy of silence’. In fact, many leading scholars of biology, psychology, anthropology, demography, and statistics devised and promoted racist classifications and advocated for eugenics (Farber, 2011; Kevles, 1985; Redvaldsen, 2024; Sear, 2021). Prominent Western scientists played a key role in reifying the idea of biological ‘race’, which in turn fostered unfair and prejudicial systems (Guevara et al., 2023). From the eighteenth to the twentieth centuries, many eminent scholars, including Carl Linnaeus, George Cuvier, Samuel George Morton, Francis Galton, Thomas Huxley, and Carlton Coon, provided biological justifications for racial hierarchies that placed White people at the top (Farber, 2011; Painter, 2010; Sussman, 2014). The histories of anthropology, biology, psychology, demography, and statistics are all tarnished with racism (G. Richards, 2012), including the propagation of eugenics by those who designed groundbreaking statistical tests still used today (Kevles, 1985; O’Connor & Robbins, 2024; Redvaldsen, 2024; Sear, 2021). Faulty interpretation of science also led to the eugenics movement and to restrictions on immigration, marriage, and parenthood in many countries, culminating in the genocides of Nazi Germany (Fredrickson, 2002/2015). As described above, scientific racism continues to this day (Sear, 2022, 2023; Lala & Feldman, 2024).

A serious problem here is the failure of scholars to openly acknowledge their disciplines’ histories of scientific racism for fear of putting students off their fields. This concern is not entirely ill-founded; students in the social sciences have been deterred from studying evolution because of its historical association with racism and eugenics (Brown et al., 2011). However, the greater danger is that, by sweeping any ‘ugly’ past of their field under the rug, scholars may alienate students from minoritized and disadvantaged backgrounds. This effect is likely to become stronger as students become more aware of the history of their field, and it may inadvertently give the message that racist historical episodes are regarded by contemporary scientists as unimportant. Acknowledging that ‘race’ and racism are deep structural problems within academia is a key recommendation made by experts on institutional racism (Arday & Mirza, 2018; Sian, 2019).

Academics sometimes respond defensively to the accusation that their field’s founders or leaders were racist using excuses and denial, which, at an institutional level, is a trait analogous to ‘white fragility’ (DiAngelo, 2018). Both Charles Darwin and Ronald Fisher have recently been subject to this apologism. For instance, when anthropologist Agustín Fuentes (2021, p. 769) marked the 150th anniversary of publication of Darwin’s *The Descent of Man* by characterizing Darwin’s writing as ‘a racist and sexist view of humanity’, it provoked strong negative reactions from several prominent biologists (e.g. Kutschera, 2021; Whiten et al., 2021). Likewise, when, in 2020, the leading academic society for evolutionary biology, the Society for the Study of Evolution (SSE) (2020) renamed its ‘R. A. Fisher Prize’, acknowledging that the British evolutionary biologist Ronald Fisher’s ‘racist views and promotion of eugenics … were relentless, harmful and unsupported by scientific evidence’, 10 past presidents and vice presidents of the Society apparently signed a letter objecting to this assessment (Coyne, 2021). Diogo et al. (2023) warned against the deification of the founders of the evolutionary sciences and the idolization of individuals who held, and/or actively promoted, prejudiced views. Academics must find a way to acknowledge, and build on, the valuable scientific contributions of key scientists while reflecting openly and honestly on the highly damaging impact of these scientists’ words, actions, and prejudices.

Historians have interpreted Darwin’s criticism of slavery, his advocacy of a monogenic (single origin) rather than polygenic (multiple origin) view of human ancestry, and his awareness of the impact that a Western education could have on the personal development of individuals from other societies as evidence that Darwin was a progressive thinker in the British Victorian age (Desmond & Moore, 2011; R. J. Richards, 1989). Although this may be true, in *The Descent of Man* (Chapter VII) and elsewhere, Darwin still explicitly partitioned humanity into distinct ‘races’, equating these with subspecies, maintaining that the White ‘race’ was superior to other ‘races’ and regularly distinguishing ‘higher races’ (or ‘modern civilized nations’) from ‘lower races’ (‘lowest savages, ‘Barbarians’), with the latter deemed to possess smaller brains and lower intellect (e.g. Darwin, 1871/1981, pp. 216, 227–228, 234, 237–239, 247; also documented in Peterson, 2024, sable 22.1; Markel, 2024, pp. 257–259). Noting that philosophers distinguish between two components of racism – *antipathy* and *inferiorization* – historian Erik Peterson (2024) documents extensive evidence of the latter in Darwin’s belief that ‘savages’ were intellectually and morally inferior and destined to be supplanted by ‘civilized men’. This position incontrovertibly meets contemporary academic definitions of scientific racism and places Darwin among the most influential propagators of racist views. Peterson (2024, p. 260) describes how, following *The Descent of Man*, ‘scientific texts adopted Darwinian language to justify racist assumptions with astonishing rapidity’. It is not helpful to claim that ‘Darwin was neither a racist nor a sexist’ (Kutschera, 2021) on the grounds that he was broad-minded or progressive in some regards. Nor should founders of academic fields avoid criticism if their racist views were the norm during their own lifetimes or if the proliferation of such views might have taken place even without their specific involvement. As Peterson (2024, p. 259) points out, to suggest that Darwin was a just ‘a man of his time’ implies that there were, at the time, no prominent dissenting voices but ‘there were such voices, and Darwin knew about them’.

Apologists often equate racism with simply holding prejudicial views (while refuting that their ‘hero’ held such prejudices), a definition at odds with contemporary scholarship but alarmingly consistent with the definition embraced by White supremacists, who also often deny being racist. Reliance on this white-supremacist definition is what underlies the mistaken but frequent inference that historical figures can be cleared of racism because they held a close or respectful association with a person of colour (e.g. Darwin’s friendship with Orundellico from Tierra del Fuego), which is evocative of the ‘I’m not racist – some of my best friends are Black’ trope (DiAngelo, 2018). Some academics can be sufficiently determined to find their heroes ‘not racist’ that they are willing to redefine racism, and reinterpret history (Peterson, 2024), to support their claims. Evolutionary biologist Jerry Coyne (2021) attributed the SSE’s renaming of the Fisher Prize to ‘wokeness’, asserting that Fisher ‘promoted eugenics, though not of the racist variety but the classist variety’, and implying that we should overlook any past transgressions because ‘none of his recommendations was ever made into policy’. In fact, Fisher’s scientific racism, when judged by standard academic definitions, is perfectly clear from his own writings. For example, when he declined to sign the UNESCO statement on ‘race’, Fisher (1952, cited in Bodmer et al., 2021) wrote, ‘available scientific knowledge provides a firm basis for believing that the groups of mankind differ in their innate capacity for intellectual and emotional development seeing that such groups do differ undoubtedly in a very large number of their genes’, an assertion that does not have, and has never had, scientific support (Lewontin, 1972; Rosenberg et al., 2002, 2005).

Apologism is surely connected with attempts to limit the reputational damage to the field, sometimes explicitly so. Concerning Fuentes’ review (Fuentes, 2021) of Darwin’s *Descent of Man*, Andrew Whiten and colleagues (2021) wrote, ‘We fear that Fuentes’ vituperative exposition will encourage a spectrum of anti-evolution voices and damage prospects for an expanded, more gender and ethnically diverse new generation of evolutionary scientists.’ Academic authorities on racism, anti-racist actions, and inclusive teaching initiatives regard these fears as misguided (Advance HE Anti-racist Curriculum Project Guide, 2020; DiAngelo, 2018; Reid, 2021). Apologism is perfectly transparent to a constituency of politically aware students and can alienate students of colour from entering or remaining within the focal field. Initiatives to decolonize curricula have advocated moving away from a culture of denial and exclusion towards acknowledgement and contextualization of historical transgressions (e.g. Shahjahan et al., 2022). Open appraisal of such behaviour, while giving voice to alternative perspectives, including those of the minoritized, marginalized, or excluded, is a central pillar of anti-racism, inclusive teaching, and curriculum decolonization.

**Solution:** *The historical background to academic fields, including racist histories, should be openly acknowledged and appropriately contextualized.*

## 4. Conclusions

The history of the evolutionary human sciences is closely intertwined with a history of racism and other forms of prejudice within the core disciplines of biology, anthropology, psychology, and demography. Although the majority of researchers in the contemporary subfields of the evolutionary human sciences reject racist ideas, the presence of racist pseudoscience on the periphery of this discipline requires urgent attention. The scientific evidence is clear: human genetic data do not support the concept of ‘races’. Yet, even within academia, several barriers prevent this important message from receiving wider recognition and acknowledgement. Genetic determinism, an over-emphasis on genetic inheritance, and the naturalistic fallacy all work together to reinforce the false view that variation among racialized groups is ‘fixed’ and ‘inborn’. These impediments also undermine the correct view that *inequitable niches* (i.e. highly distinctive experiences of the world) have been, and continue to be, created by racism and discrimination, and that they are actively maintained through the legacies of inherited norms and institutions, inherited wealth and power, inherited values and traditions, and inherited environments that vary in their amenities and opportunities (Ivey Henry et al., 2023; Lala & Feldman, 2024). Incorrect understanding of ‘race’ and racism is further enabled by failure to teach the relevant science and by apologism for the racism expounded by founding scientists in this field. Addressing racism requires moving away from a culture of denial and exclusion towards honest acknowledgement and contextualization of historical transgressions.

Although we have recommended brief ‘solutions’ to each identified ‘impediment’, making recommendations will not in itself bring about change. Meaningful progress in countering racist pseudoscience will require the incorporation of these recommendations as ‘community-endorsed learning objectives’ in modernized curricula for relevant (particularly, but not exclusively, biology) high school and undergraduate classes (Dougherty, 2009; Hennessey & Freeman, 2024). We encourage scholars of the evolutionary human sciences to be active in supporting this objective, and those with authority over school, college, and university curricula to implement these recommendations.

## Data Availability

NA.

## Research transparency and reproducibility

No new data are presented.
