# Impediments to countering racist pseudoscience - CORRIGENDUM

**DOI:** 10.1017/ehs.2025.10028

**Published:** 2025-11-18

**Authors:** Kevin N. Lala, Gillian Brown, Kalyani Twyman, Marcus W. Feldman

The above article was published with errors in the text which are listed below.

The author apologises for these errors.

Page 4, Table 1 Caption “Impediments to racist pseudoscience and potential practical solutions” should read “Impediments to countering racist pseudoscience and potential practical solutions”.

Page 4, Left Column Heading “Impediments to racist pseudoscience and potential practical solutions” should read “Impediments to countering racist pseudoscience and potential practical solutions”.
